# A straightforward edge centrality concept derived from generalizing degree and strength

**DOI:** 10.1038/s41598-022-08254-5

**Published:** 2022-03-15

**Authors:** Timo Bröhl, Klaus Lehnertz

**Affiliations:** 1 Department of Epileptology, University of Bonn Medical Centre, Venusberg Campus 1, 53127 Bonn, Germany; 2 Helmholtz-Institute for Radiation and Nuclear Physics, University of Bonn, Nussallee 14–16, 53115 Bonn, Germany; 3 Interdisciplinary Center for Complex Systems, University of Bonn, Brühler Straße 7, 53175 Bonn, Germany

**Keywords:** Physics, Statistical physics, thermodynamics and nonlinear dynamics, Complex networks

## Abstract

Vertex degree—the number of edges that are incident to a vertex—is a fundamental concept in network theory. It is the historically first and conceptually simplest centrality concept to rate the importance of a vertex for a network’s structure and dynamics. Unlike many other centrality concepts, for which joint metrics have been proposed for both vertices and edges, by now there is no concept for an edge centrality analogous to vertex degree. Here, we propose such a concept—termed nearest-neighbor edge centrality—and demonstrate its suitability for a non-redundant identification of central edges in paradigmatic network models as well as in real-world networks from various scientific domains.

## Introduction

Complex network approaches have been repeatedly shown to provide deeper insights into structure and dynamics of spatially extended complex systems in diverse areas of science^1–9^. In many natural and man-made networked systems, access to the underlying coupling structure may be restricted or even impossible. Nevertheless, in such cases can the system be described by an interaction network with vertices representing subsystems or elementary units and edges representing interactions between them. This ansatz has been successfully applied e.g. in the study of (functional) brain networks^10^, climate networks^11,12^, protein-protein interactions^13^, gene interactions^14^, plant-pollinator interactions^15,16^, food-webs^17^, or communication and social networks^18,19^.

In order to further improve understanding and control of interaction networks, the identification of key network constituents and a characterization of their importance for a network’s structure and dynamics is highly relevant^20–27^. There are different concepts and a growing number of metrics—such as centralities—that allow one to characterize the role of network vertices for structure and dynamics^28^. There are by now, however, only a few metrics for edge centrality. Many of them center around the concept of betweenness centrality^29–35^, other make use of the concept of bridging^36–39^ or are based on the spectrum of the network’s Laplacian^40,41^. We have recently introduced modifications of closeness and eigenvector centrality concepts for vertices to those for edges and demonstrated that these edge centralities provide additional information about the network constituents for various topologies^42^.

The aforementioned joint centrality concepts for vertices and edges can be classified as path-based (betweenness centrality $\mathcal {C}^{\mathrm {B}}$ and closeness centrality $\mathcal {C}^{\mathrm {C}}$) or degree/strength-based (eigenvector centrality $\mathcal {C}^{\mathrm {E}}$). Interestingly, there is by now no edge centrality concept analogous to the historically first and conceptually simplest vertex centrality^43^, namely vertex degree centrality, which is defined as the number of edges incident upon a vertex. In case of a weighted network, the corresponding vertex strength centrality^44,45^ is defined as the sum of weights of these edges. Here, we propose such an analogous edge centrality concept, which we termed nearest-neighbor edge centrality $\mathcal {C}^{\mathrm {N}}$. Using various paradigmatic network models, we illustrate this edge centrality concept and investigate possible relationships to the other aforementioned edge centrality concepts. We will then apply the novel concept to identify important edges in a commonly used benchmark model in social network analysis, in a commuter network as well as in evolving epileptic brain networks.

## Results and discussions

### Joint centrality concepts for vertices and edges

Let us briefly recall and discuss the most commonly used joint centrality concepts (betweenness centrality $\mathcal {C}^{\mathrm {B}}$, closeness centrality $\mathcal {C}^{\mathrm {C}}$, and eigenvector centrality $\mathcal {C}^{\mathrm {E}}$) for vertices and edges. We here consider binary or weighted, undirected and connected networks that consist of sets of vertices $\mathcal {V}$ and edges $\mathcal {E}$, with $V = \left| \mathcal {V}\right|$ and $E = \left| \mathcal {E}\right|$ denoting the number of vertices and edges, respectively. We do not consider self-loops or parallel edges. Centrality concepts that are based on shortest paths require the definition of “length” $d_{ij}$ of a path between vertices *i* and *j*. In a binary network, the length $d_{ij}$ of a shortest path P between vertices *i* and *j* is the number of edges along this path. We define $d_{ii} \mathrel {\mathop :}=0$ as we do not consider self-loops. In a weighted network, since an edge weight represents the strength of a connection between two vertices, we consider a path to be shorter the stronger the connections along this path are. Consequently, we relate $d_{ij}$ of path P between vertices *i* and *j* to the sum of the inverse weights of edges along this path^43^. A shortest path can be defined as the path between two vertices for which the sum of the inverse weights of edges along this path is minimal^42^.

### Betweenness centrality

With betweenness centrality $\mathcal {C}^{\mathrm {B}}$, a network constituent is the more central the more shortest paths pass through this constituent. Vertex/edge betweenness centrality (of vertex/edge *k*) can be defined as^30,46,47^

1 $$\begin{aligned} \mathcal {C}^{\mathrm {B}}_{\mathrm {v,e}} (k)=\frac{2}{F}\sum _{i\ne j}\frac{q_{ij}(k)}{G_{ij}}, \end{aligned}$$

where $k \in \left\{ 1,\ldots ,V\right\}$, resp. $k \in \left\{ 1,\ldots ,E\right\}$, $\left\{ i,j\right\} \in \left\{ 1,\ldots ,V\right\}$, $q_{ij}(k)$ is the number of shortest paths between vertices *i* and *j* running through vertex/edge *k*, and $G_{ij}$ is the total number of shortest paths between vertices *i* and *j*. The normalization factor is $F=(V-1)(V-2)$ in case of vertices and $F=V(V-1)$ in case of edges.

With this definition (Eq. 1), $\mathcal {C}^{\mathrm {B}}_{\mathrm {v}}$ may assign disproportionately large centrality values to vertices with an arbitrary and possible very low degree (at least 2) and a neighboring vertex of degree 1, as every shortest path between the degree-1 vertex and every other vertex in the network has to traverse the vertex adjacent to the degree-1 vertex. In a similar manner will an edge between a degree-1 vertex and the adjacent vertex be assigned a disproportionately large centrality value. Apart from the normalization factor *F*, $\mathcal {C}^{\mathrm {B}}_{\mathrm {v}}$ and $\mathcal {C}^{\mathrm {B}}_{\mathrm {e}}$ are equally defined and for the latter, one also considers—contrary to intuition—the shortest paths between all vertex pairs and not between all possible pairs of edges in the network.

This of course has also the advantage of reducing computation time drastically when calculating edge betweenness centrality. Furthermore, $\mathcal {C}^{\mathrm {B}}$ does not directly depend on the distribution of edge weights in the network. It finds wide application and the concept yields distinct and non-redundant information about a network in comparison with the other centrality concepts.

### Closeness centrality

With closeness centrality $\mathcal {C}^{\mathrm {C}}$, a constituent is the more central the shorter the paths that connect this constituent to every other reachable constituent of the same type. Closeness centrality of vertex *k* is defined as^48^:

2 $$\begin{aligned} \mathcal {C}^{\mathrm {C}}_{\mathrm {v}} (k)=\frac{V-1}{\sum _{i}{d_{ik}}}, \end{aligned}$$

with $(k,i) \in \left\{ 1,\ldots ,V\right\}$. Closeness centrality of edge *k* between vertices *a* and *b* can the be defined as^42^:

3 $$\begin{aligned} \begin{aligned} \mathcal {C}^{\mathrm {C}}_{\mathrm {e}} (k)&=\frac{E-1}{\sum _{i}{(d_{ia}+d_{ib})}} =\frac{E-1}{\frac{1}{\mathcal {C}^\mathrm{C}_\mathrm{v}(a)}+\frac{1}{\mathcal {C}^\mathrm{C}_\mathrm{v}(b)}}\\&=(E-1)\frac{\mathcal {C}^\mathrm{C}_\mathrm{v}(a)\mathcal {C}^\mathrm{C}_\mathrm{v}(b)}{\mathcal {C}^\mathrm{C}_\mathrm{v}(a)+\mathcal {C}^\mathrm{C}_\mathrm{v}(b)}, \end{aligned} \end{aligned}$$

with $$k \in \left\{ 1,\ldots ,E\right\} $$ and $$(a,b,i)\in \{1,\dots ,V\}$$.

Closeness centrality is the only centrality concept that directly depends on the path structure in a network as well as on the distribution of edge weights. The concept mostly finds application in network studies that model some kind of information flow. Nevertheless, due to its definition, $\mathcal {C}^{\mathrm {C}}$ lacks applicability regarding networks with disconnected components^49,50^.

### Eigenvector centrality

With eigenvector centrality $\mathcal {C}^{\mathrm {E}}$, a network constituent is central if its adjacent constituents of the same type are also central. Eigenvector centrality $\mathcal {C}^{\mathrm {E}}$ of vertex^51^ or edge^42^ *k* is defined as the *k*th entry of the eigenvector $\mathbf {v}$ corresponding to the dominant eigenvalue $\lambda _{\max }$ of matrix $\mathbf{M}$, which can be derived from the eigenvector equation $\mathbf{M}\mathbf {v}=\lambda \mathbf {v}$ using the power iteration method:

4 $$\begin{aligned} \mathcal {C}^{\mathrm {E}}_{\mathrm {v,e}} (k)=\frac{1}{\lambda _{\max }}\sum _{l}^{}M_{kl}\,\mathcal {C}^{\mathrm {E}}_{\mathrm {v,e}} (l). \end{aligned}$$

In case of vertices, $\left\{ k,l\right\} \in \mathcal {V}$ and $\mathbf{M}$ denotes the adjacency matrix $\mathbf{A}^\mathrm{(v)} \in \left\{ 0,1\right\} ^{V \times V}$ of a binary network, with $A^\mathrm{(v)}_{kl}=1$ if there is an edge between vertices *k* and *l*, and 0 otherwise. In case of a weighted network, $\mathbf{M}$ denotes the weight matrix $\mathbf{W}^\mathrm{(v)} \in \mathbb {R}_+^{V \times V}$, with $W^\mathrm{(v)}_{kl}$ denoting the weight of an edge between vertices *k* and *l*. In a binary network, the degree $\kappa _k$ of vertex *k* is defined as the number of its neighbors ($\kappa _k \mathrel {\mathop :}=\sum _{j} A^\mathrm{(v)}_{kj}$). Its weighted counterpart is the strength $s_k \mathrel {\mathop :}=\sum _{j} W^\mathrm{(v)}_{kj}$. We define $A^\mathrm{(v)}_{kk} \mathrel {\mathop :}=0 \,\forall \, k$ and $W^\mathrm{(v)}_{kk} \mathrel {\mathop :}=0 \,\forall \, k$ with $k\in \left\{ 1,\ldots ,V\right\}$.

In case of edges, $\left\{ k,l\right\} \in \mathcal {E}$ and $\mathbf{M}$ denotes the edge adjacency matrix $\mathbf{A}^\mathrm{(e)} \in \left\{ 0,1\right\} ^{E \times E}$ of a binary network, with $A^\mathrm{(e)}_{kl}=1$ if edges *k* and *l* are connected to a same vertex, and 0 otherwise. In case of a weighted network, $\mathbf{M}$ denotes the weight matrix $\mathbf{W}^\mathrm{(e)} \in \mathbb {R}_+^{E \times E}$ whose entries $W^\mathrm{(e)}_{kl}$ are assigned the average weight of edges *k* and *l* if these edges are connected to a same vertex, and 0 otherwise. As above, we define $A^\mathrm{(e)}_{kk} \mathrel {\mathop :}=0 \,\forall \, k$ and $W^\mathrm{(e)}_{kk} \mathrel {\mathop :}=0 \,\forall \, k$ with $k\in \left\{ 1,\ldots ,E\right\}$.

Eigenvector centrality also presents with some limitations. Much like for closeness centrality, $\mathcal {C}^{\mathrm {E}}$ lacks applicability to networks with disconnected components. Depending on the network structure, one might encounter weight distributions that decrease exponentially with increasing degree. In this case most of the constituents will be assigned centrality values close to zero and, therefore, the importance of the constituents may insufficiently be quantified.

However, compared to $\mathcal {C}^{\mathrm {B}}$ and $\mathcal {C}^{\mathrm {C}}$, $\mathcal {C}^{\mathrm {E}}$ is the only centrality concept based on spectral properties of the (weighted) adjacency matrix. While the path-based concepts $\mathcal {C}^{\mathrm {B}}$ and $\mathcal {C}^{\mathrm {C}}$ only indirectly consider the network as a whole to identify shortest paths, the strength-based concept $\mathcal {C}^{\mathrm {E}}$ considers the structure of the total network in a gradual but direct manner.

### Nearest-neighbor edge centrality

With the concept of strength centrality, a vertex is the more central the stronger the connections to adjacent vertices are. The strength (or strength centrality) of vertex *k* reads:

5 $$\begin{aligned} \mathcal {C}^{\mathrm {S}}_{\mathrm {v}} (k)= s_k =\sum _{j} W^\mathrm{(v)}_{kj}, \end{aligned}$$

with $(k,j) \in \left\{ 1,\ldots ,V\right\}$ and the weight matrix element $W^\mathrm{(v)}_{kj}$ (see above). Analogously the degree $\mathcal {C}^{\mathrm {D}}_{\mathrm {v}} (k)$ of a vertex *k* is defined as sum of adjacent vertices to vertex *k*. The concept on its own is limited to the total level of involvement of a vertex in the network and does not take into account intrinsic properties of a vertex (as there exist no such properties). Instead it considers an intrinsic property of edges connected to a vertex, namely the edge weights. Moreover, it does not take into account the number of adjacent vertices, which has been described as a main feature in Freeman’s centrality metrics^43^. Aiming to derive a comparably simple and straightforward definition of edge centrality, one naively could use the edge weight itself. This would give, however, no perspective of the edges’ role in a network, as an edge weight has no direct relation to the network’s structure. Furthermore, such an edge centrality would not represent an analogue to the degree/strength of a vertex. To achieve just that, an edge centrality would have to depend on intrinsic properties of the two vertices connected by an edge. As there is no intrinsic vertex property, we here resort to the vertices’ degrees/strengths to derive a ‘strength’-related centrality concept for edges. We consider an edge to be more central the larger its weight and the more similar and the higher the strengths of the vertices which are connected by that edge. For a binary network, we define nearest-neighbor edge centrality $\mathcal {C}^{\mathrm {N}}_{\mathrm {e}}$ of an edge *k* between vertices *a* and *b* as:

6 $$\begin{aligned} \mathcal {C}^{\mathrm {N}}_{\mathrm {e}} (k)=\frac{\mathcal {C}^{\mathrm {D}}_{\mathrm {v}} (a)+\mathcal {C}^{\mathrm {D}}_{\mathrm {v}} (b)-2}{|\mathcal {C}^{\mathrm {D}}_{\mathrm {v}} (a)-\mathcal {C}^{\mathrm {D}}_{\mathrm {v}} (b)|+1}\;, \end{aligned}$$

where $$k \in \left\{ 1,\ldots ,E\right\} $$ and $$(a,b)\in \{1,\dots ,V\}$$.

Analogously, for a weighted network we define:

7 $$\begin{aligned} \mathcal {C}^{\mathrm {N}}_{\mathrm {e}} (k)=\frac{\mathcal {C}^{\mathrm {S}}_{\mathrm {v}} (a)+\mathcal {C}^{\mathrm {S}}_{\mathrm {v}} (b)-2w_k}{|\mathcal {C}^{\mathrm {S}}_{\mathrm {v}} (a)-\mathcal {C}^{\mathrm {S}}_{\mathrm {v}} (b)|+1}\;w_k, \end{aligned}$$

where $k \in \left\{ 1,\ldots ,E\right\}$ and $(a,b)\in \{1,\dots ,V\}$, and $w_k$ denotes the weight of edge *k* connecting vertices *a* and *b*.

The numerator of the fraction of Eq. 7 (Eq. 6) captures the ‘strength’ (‘degree’) of the edge, as it effectively describes the sum of weights of adjacent edges (sum of adjacent edges)—edges that share a vertex. The denominator represents the difference of the strengths of the two vertices connected by the edge. Hence, an edge is the more central the larger the weights of its adjacent edges are and the more symmetrical these edge weights are distributed between the two vertices. We therefore define an edge to be more central if it is connected to vertices that are both strongly connected in the network than an edge that is connected to one very strongly connected vertex and one weakly connected vertex (e.g., an edge as one of many edges connected to a hub). Furthermore, the weight of the edge itself contributes to its centrality. This compensates for the fact that even overall weakly connected vertices, with possibly high degrees but low strength, can also be connected by a highly central edge. Overall, nearest-neighbor edge centrality is independent of the network’s topology and size and is solely based on local network characteristics. An additional normalization factor $(\frac{1}{2(V-2)})$ can be considered when aiming at a comparison with other edge centrality concepts, since established edge centrality concepts (e.g., $\mathcal {C}^{\mathrm {B}}_{\mathrm {e}}$) are also normalized with respect to the total number of vertices.

### Comparison with other edge centralities

**Figure 1:**
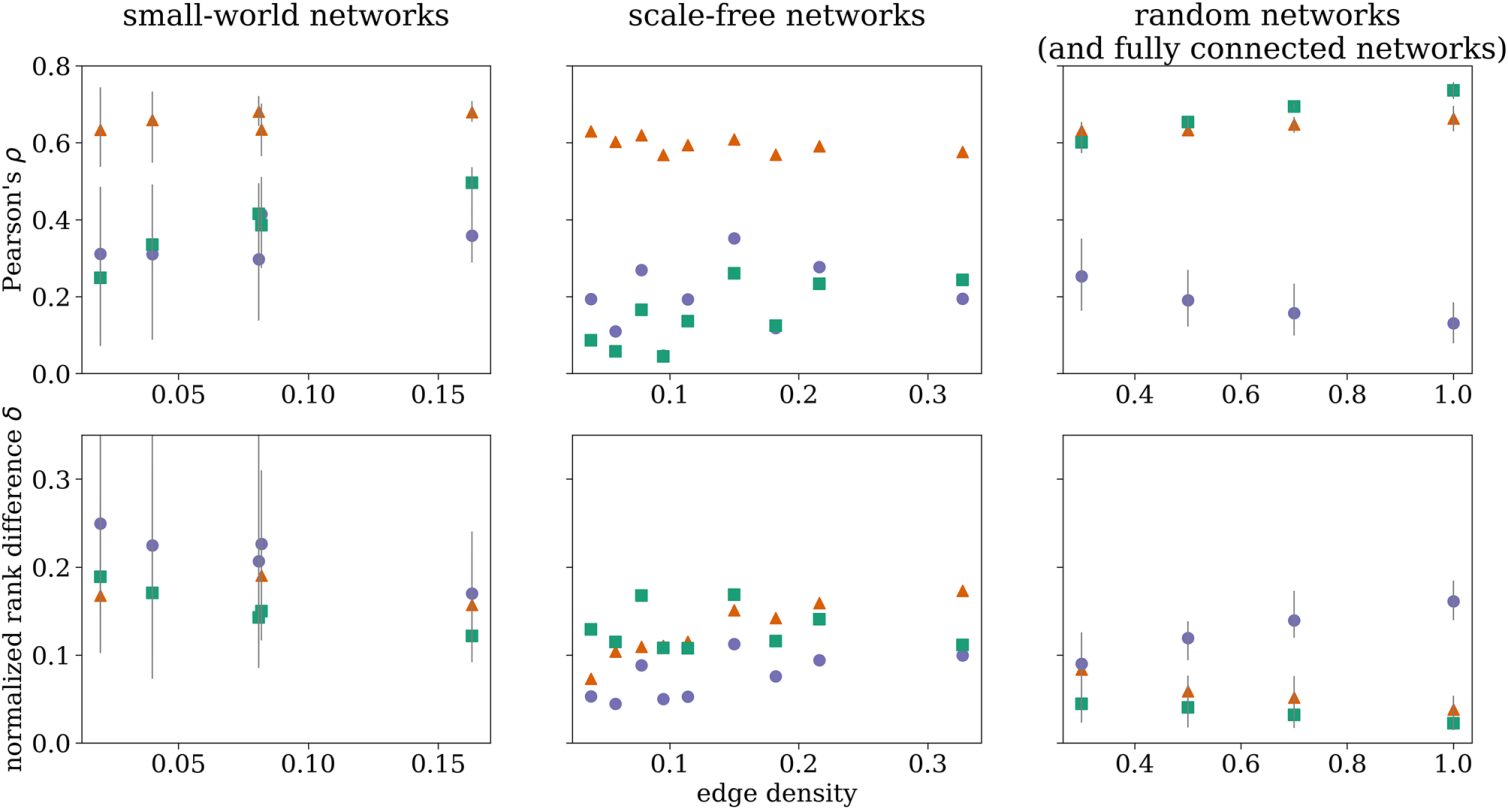
Nearest-neighbor edge centrality provides non-redundant information about edges in paradigmatic network models. Correlations (Pearson’s $\rho$) of edge ranks (top) and normalized rank differences $\delta$ (bottom) obtained with the different centrality metrics for the investigated network topologies and edge densities. Means and ranges (lengths of error bars) obtained from 100 realizations of the network topologies. Different colors encode pairs of edge centralities used for correlation analyses and analysis of normalized rank differences: orange triangle—($\mathcal {C}^{\mathrm {N}}_{\mathrm {e}}$,$\mathcal {C}^{\mathrm {B}}_{\mathrm {e}}$), purple circle—($\mathcal {C}^{\mathrm {N}}_{\mathrm {e}}$,$\mathcal {C}^{\mathrm {C}}_{\mathrm {e}}$), and green square—($\mathcal {C}^{\mathrm {N}}_{\mathrm {e}}$,$\mathcal {C}^{\mathrm {E}}_{\mathrm {e}}$). Note that for scale-free networks error bars are smaller than symbol size.

We begin by addressing the question whether our introduced centrality concept provides non-redundant information about edges in weighted networks when compared to other edge centrality concepts. To this end, we investigate paradigmatic network models with different sizes $V \in \left\{ 50, 100, 200\right\}$, perform correlation analyses of edge ranks obtained with the different centrality metrics, and investigate the normalized rank difference $\delta =(\mathrm {rank}^\mathrm {N}(\Omega )-\mathrm {rank}^\mathrm {\bullet }(\Omega ))/E=(1-\mathrm {rank}^\mathrm {\bullet }(\Omega ))/E$, where $\Omega$ denotes the most important edge as identified with $\mathcal {C}^{\mathrm {N}}_{\mathrm {e}}$, and $\bullet \in \{\mathrm {B},\mathrm {C},\mathrm {E}\}$. If the nearest-neighbor centrality concept identifies the same edge as most important (rank 1) as the centrality concept we compare it to (betweenness, closeness, eigenvector) , $\delta$ will vanish.

We consider small-world networks^52^ (with rewiring probabilities $p_r \in \left\{ 0.01, 0.1, 0.2, 0.3\right\}$), scale-free networks^53^ (parameter of attachment $m\in \left\{ 4, 6, 10\right\}$), random networks^54,55^ (with edge creation probabilities $p_c \in \left\{ 0.3, 0.5, 0.7\right\}$), and fully connected networks, for each of which we generated 100 realizations. For each realization of these weighted networks, we draw weights from some distribution, and in the case of equal centrality values (for a given centrality concept), we rank in the order of appearance. All networks are undirected and connected, contain no self-loops and no multiple edges.

Figure 1 summarizes our findings for the aforementioned analyses with weights drawn from the uniform distribution $\mathcal {U}(0,1)$. We obtained similar findings for weights drawn from other distributions (Gaussian, Gumbel with different locations of the mode). In general, we observe a wide range of correlation values ($\rho \in \left[ 0.0,0.8\right]$) and these vary for different network topology as well as for different edge densities. We find overall highest correlation values ($0.7 < \rho \le 0.8$) for a comparison with $\mathcal {C}^{\mathrm {E}}_{\mathrm {e}}$ which, however, is to be expected since both these edge centralities strongly depend on the weight distribution of the edges that are adjacent to the edge under investigation. For small-world and scale-free networks, we observe highest correlation values when comparing with $\mathcal {C}^{\mathrm {B}}_{\mathrm {e}}$, which may be related to path structure properties that are characteristic for these topologies: short-cuts and bottlenecks. In case of the most important edge as identified with $\mathcal {C}^{\mathrm {N}}_{\mathrm {e}}$, we observe $\mathcal {C}^{\mathrm {C}}_{\mathrm {e}}$ and $\mathcal {C}^{\mathrm {E}}_{\mathrm {e}}$ to yield more similar ranks than $\mathcal {C}^{\mathrm {B}}_{\mathrm {e}}$. Based on the definitions of $\mathcal {C}^{\mathrm {N}}_{\mathrm {e}}$, $\mathcal {C}^{\mathrm {C}}_{\mathrm {e}}$ and $\mathcal {C}^{\mathrm {E}}_{\mathrm {e}}$, similarities—especially in identifying the most important edge—are to be expected. Nevertheless, the most important edge as identified with $\mathcal {C}^{\mathrm {N}}_{\mathrm {e}}$ does not coincide with the most important edge as identified with the other centrality concepts. Furthermore, we observe very few concordances between central edges (up to rank 10) as identified with $\mathcal {C}^{\mathrm {N}}_{\mathrm {e}}$ and those identified with one of the other three centralities (data not shown). In case of small-world networks and weights drawn from a Gumbel distribution with a location of the mode around 0.2, we observe highest concordance. Here, in approx. $37\%$ of realisations, $\mathcal {C}^{\mathrm {N}}_{\mathrm {e}}$ and $\mathcal {C}^{\mathrm {E}}_{\mathrm {e}}$ identify the same most important edge. However, over all realizations, the concordance rate between any edge from the top 10 ranking based on $\mathcal {C}^{\mathrm {N}}_{\mathrm {e}}$ with any edge from the top 10 ranking based on $\mathcal {C}^{\mathrm {B}}_{\mathrm {e}}$, $\mathcal {C}^{\mathrm {C}}_{\mathrm {e}}$ or $\mathcal {C}^{\mathrm {E}}_{\mathrm {e}}$ is less or equal $5\%$. Taken together, central edges identified with $\mathcal {C}^{\mathrm {N}}_{\mathrm {e}}$ are not assigned the same rank when identified with $\mathcal {C}^{\mathrm {B}}_{\mathrm {e}}$, $\mathcal {C}^{\mathrm {E}}_{\mathrm {e}}$ or $\mathcal {C}^{\mathrm {C}}_{\mathrm {e}}$, and are also not assigned a rank close to it.

Summarizing these findings, we conclude our novel centrality concept to provide non-redundant information about network edges when compared to other edge centrality concepts.

### Identifying important edges in real-world networks

We next demonstrate the utility of the proposed edge centrality concept for understanding which edges are important in real-world networks. We here focus on Zachary’s karate club network^56^, which is a commonly used benchmark model in social network analysis, on a commuter network^57–59^, and on evolving epileptic brain networks^60–62^. We regard an edge with the highest centrality value as most important and the one with the lowest centrality value as least important. In the case of equal centrality values, we rank in order of appearance.

### Zachary’s karate club network

**Figure 2:**
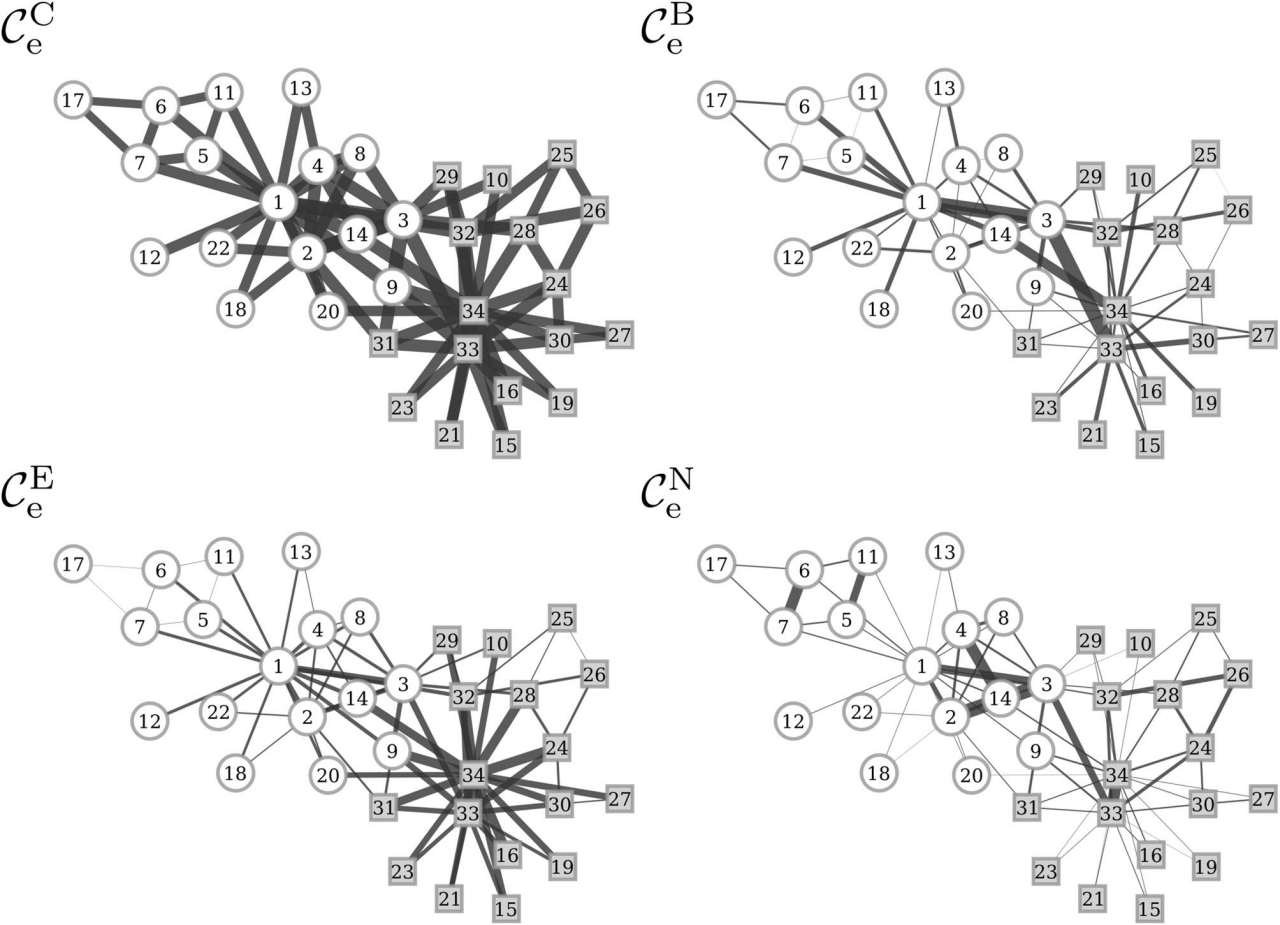
Key interactions in Zachary’s karate club network. Interactions identified with different edge centrality concepts (edge closeness centrality $\mathcal {C}^{\mathrm {C}}_{\mathrm {e}}$; edge eigenvector centrality $\mathcal {C}^{\mathrm {E}}_{\mathrm {e}}$; edge betweenness centrality $\mathcal {C}^{\mathrm {B}}_{\mathrm {e}}$; nearest-neighbor edge centrality $\mathcal {C}^{\mathrm {N}}_{\mathrm {e}}$). Edge importance is encoded as line thickness and numbered vertices represent the members of the club. The two factions into which the club split during the course of the study are indicated by circles [the club instructor’s group (vertex 1)] and squares [the club administrator’s group (vertex 34)].

The network consists of 34 persons (vertices) whose interactions (78 weighted edges) have been carefully investigated over a period of three years. Shortly after Zachary finished his research, the karate club split into two smaller groups. This was due to a conflict and disagreements between the club’s instructor (vertex 1) and administrator (vertex 34) regarding the prices of karate lessons. This ultimately resulted in the instructor’s leaving and launching a new club, taking about a half of the original club’s members with him. The fission was to 97 % correctly predicted by Zachary based on his observations regarding social interactions.

The conflict between the two individuals (instructor and administrator) was carried out on a much larger social structure, with each individual trying to win as many students for their cause. As neither the instructor nor the administrator necessarily had intensive relationships to each and every student, individual opinion formation was based on much more complex social structure modeled by the network. Furthermore, the instructor and the administrator had no direct interactions with each other and an exchange of information between them relied on close intermediaries. Word of mouth, popular students, or specific relationships (bottlenecks) might be of vital importance in this context. Thus it is essential to identify key interactions between individuals that very possibly enabled the observed fission within the club. Figure 2 depicts these key interactions that we identified with different edge centrality concepts.

With edge closeness centrality $\mathcal {C}^{\mathrm {C}}_{\mathrm {e}}$, we obtained a very narrow distribution of centrality values, which does not allow a visual identification of key interactions. This can be generally explained by the fact that any vertex in this rather small network is comparably close to every other vertex.

With edge eigenvector centrality $\mathcal {C}^{\mathrm {E}}_{\mathrm {e}}$, we identify edges with the highest centrality values to connect vertices 33 and 34 (the administrator and a close student of his) with many other vertices. Other arguably important individuals, represented by vertices 1, 3, 9 and 14, are also connected via some few high-centrality edges, however, much less than the ones observed around the hubs (i.e., vertices 33 and 34). Eigenvector centrality is closely related to strength centrality and becomes almost indistinguishable from the latter in case of small networks. Hence, it can be expected to observe key interactions as the high-centrality edges that connect the high-degree/high-strength vertices (1, 33, and 34) or as those that connect other vertices (namely vertices 3, 9, and 14) to the aforementioned vertices.

With edge betweenness centrality $\mathcal {C}^{\mathrm {B}}_{\mathrm {e}}$, many edges with comparably high centrality values connect vertex 1 (the instructor) to other vertices. Edges with highest centrality values, however, connect vertices 1 and 3, 3 and 33, as well as vertices 34 and 14. This can easily be understood since betweenness centrality is well suited to identify bottlenecks in a network. The aforementioned high-centrality edges represent some of the very few key interactions between the two parts of the network that resulted from the fission.

With nearest-neighbor edge centrality $\mathcal {C}^{\mathrm {N}}_{\mathrm {e}}$, we expect edges to reflect key interactions if they very strongly connect vertices that are equally densely integrated in the network and possibly in the two smaller groups of the network. These edges can be regarded as ‘local’ bottlenecks, possibly also coinciding with ‘global’ bottlenecks. Those local bottlenecks are edges that connect vertices 6 and 7, 5 and 11, as well as vertices 4 and 14. These edges are located in the sub-network centered around the instructor. Edges connecting vertices 1, 2, and 33 with vertex 3 can be associated with a more global bottleneck, which in part could also be identified with $\mathcal {C}^{\mathrm {B}}_{\mathrm {e}}$. The edge connecting vertices 2 and 3 has not been identified as important with $\mathcal {C}^{\mathrm {B}}_{\mathrm {e}}$, but appears to be most important using $\mathcal {C}^{\mathrm {N}}_{\mathrm {e}}$. This edge is to be associated with a larger bottleneck structure, being the path from vertex 2 to vertex 33, traversing vertex 3. Hence, vertices 2, 3, and 33 appear to be the main mediators between the club’s instructor and administrator.

The fact that $\mathcal {C}^{\mathrm {N}}_{\mathrm {e}}$ highlights both, local and global bottlenecks in the karate club network distinguishes it from $\mathcal {C}^{\mathrm {B}}_{\mathrm {e}}$. We conjecture that $\mathcal {C}^{\mathrm {N}}_{\mathrm {e}}$ can aid in an improved characterization of the path structure in complex networks.

### Commuter network of the German state North Rhine-Westphalia

The most populous state in Germany is North Rhine-Westphalia (NRW) with approx. 18 million inhabitants living on an area of more than 34,000 square kilometers. In addition to the German city-states, NRW is the most densely populated state, and commuter traffic within NRW is enormous^63^. Studying the network of commuter traffic can greatly aid to improve, e.g. understanding and control of spreading processes^57,59,64–69^. As a most recent example, we mention the spread of the corona virus SARS-CoV-2, with the urban district Heinsberg being one of the pandemic’s origins in Germany.

**Figure 3:**
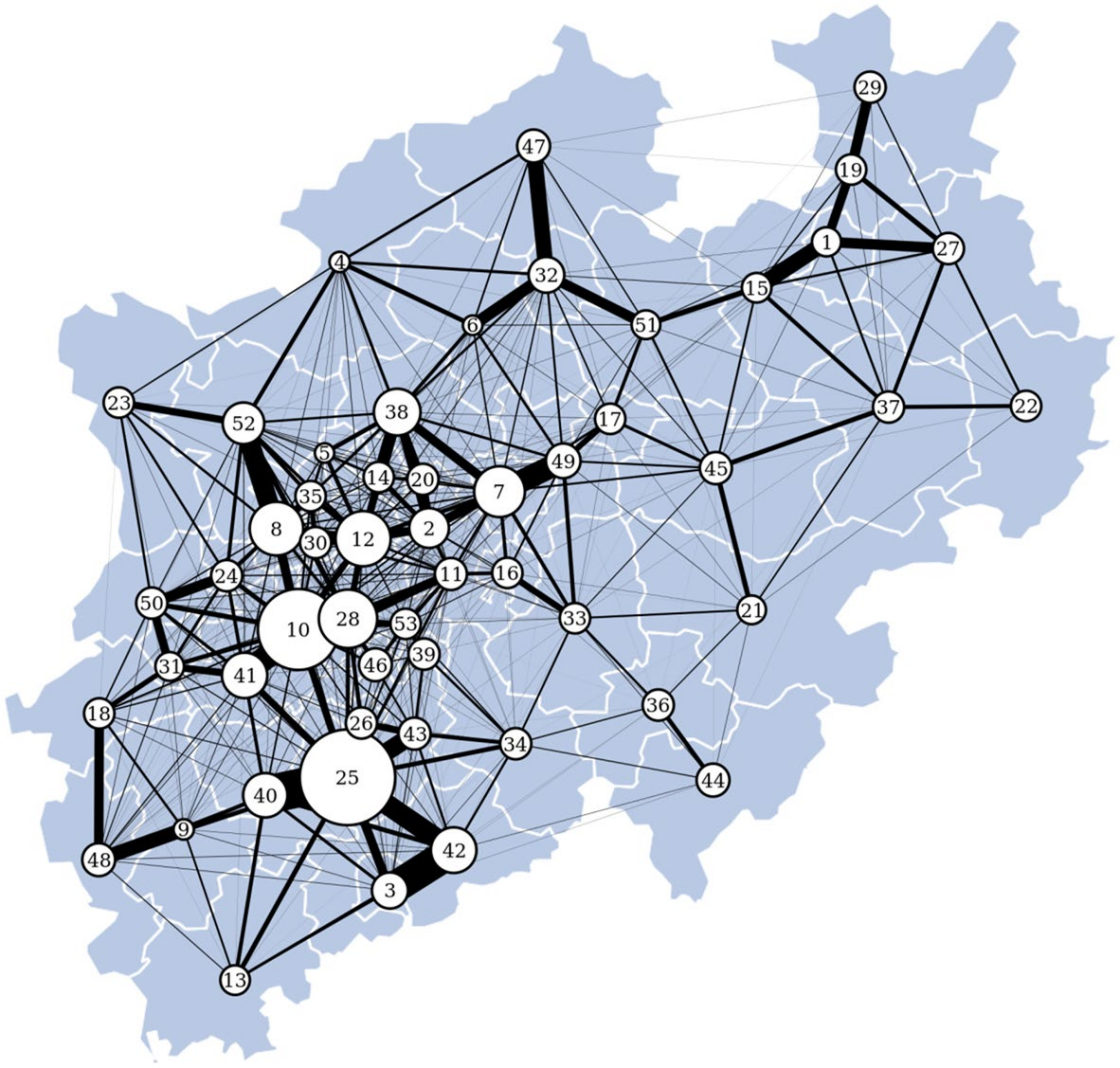
Commuter traffic network of North Rhine-Westphalia (NRW). Numbered vertices represent the rural and urban districts in NRW. Size of vertices represents their respective strength and thickness of edges represents the amount of commuter traffic between them.

**Figure 4:**
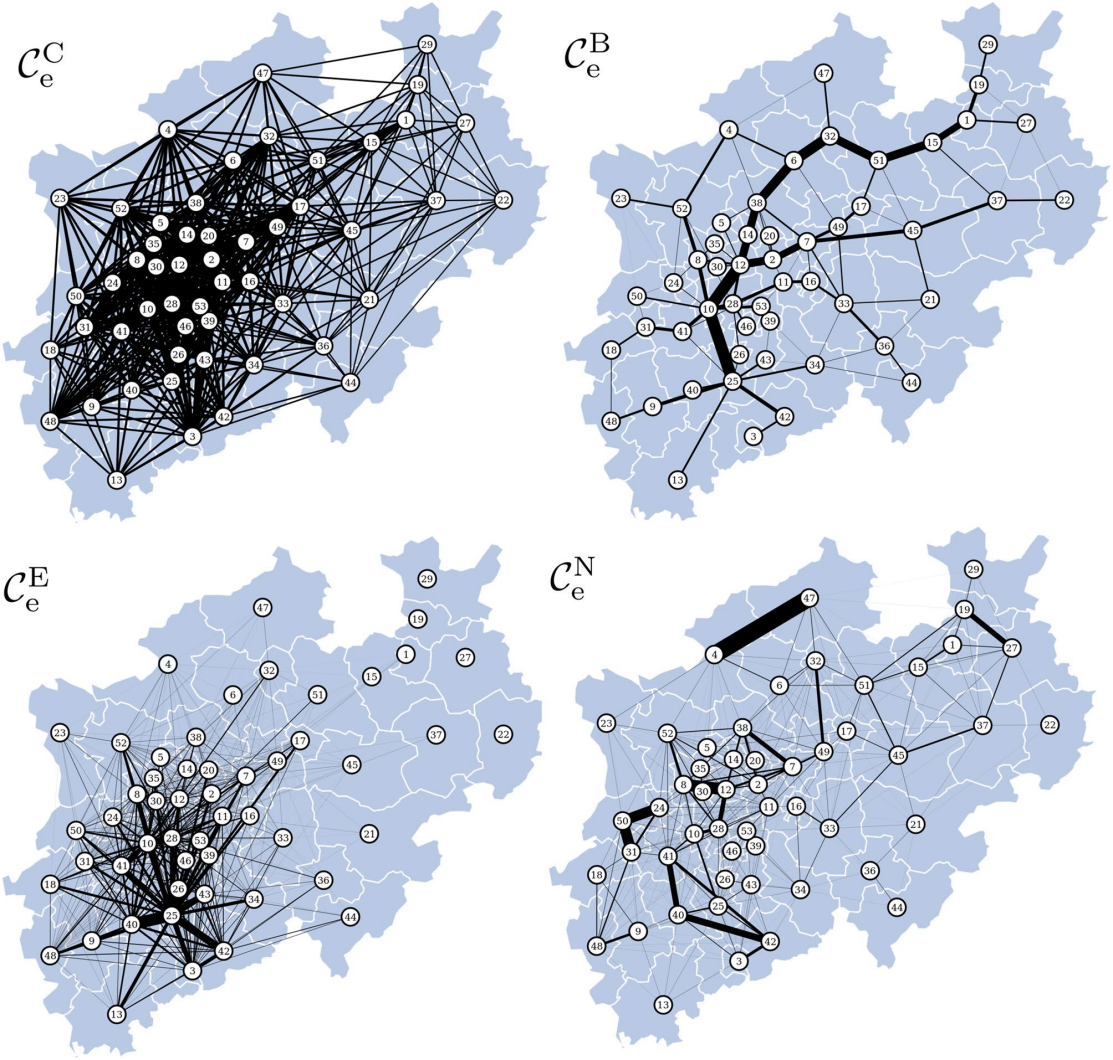
Key connections in the commuter traffic network of North Rhine-Westphalia (NRW). Connections identified with different edge centrality concepts (edge closeness centrality $\mathcal {C}^{\mathrm {C}}_{\mathrm {e}}$; edge eigenvector centrality $\mathcal {C}^{\mathrm {E}}_{\mathrm {e}}$; edge betweenness centrality $\mathcal {C}^{\mathrm {B}}_{\mathrm {e}}$; nearest-neighbor edge centrality $\mathcal {C}^{\mathrm {N}}_{\mathrm {e}}$). Numbered vertices represent the rural and urban districts in NRW and edges indicate commuter traffic between them. Edge importance is encoded as line thickness.

For our analyses, we take the rural and urban districts in NRW as vertices of the commuter traffic network (Fig. 3). We consider an edge to exist between two vertices if commuter traffic between them was recorded, and the edge weight equals the average number of commuters traveling between two vertices on a day in 2017.

Figure 4 summarizes our findings that we obtained from applying the edge centrality concepts to identify important commuter connections. Similar to Zachary’s karate club network, $\mathcal {C}^{\mathrm {C}}_{\mathrm {e}}$ does not highlight any specific connections since the distribution of edge weights is rather narrow. With $\mathcal {C}^{\mathrm {B}}_{\mathrm {e}}$, we observe a path between the north-east and south-west of NRW to be most important. It traverses geo-economically centers and population-dense districts, such as vertices 32 (Muenster), 12 (Essen), 10 (Dusseldorf) and 25 (Cologne). Interestingly enough and even though spatially close, neither of these urban districts share a common border. The identified important edges, however, spatially traverse approximately one other district and therefore would generally not be considered as long-range connections.

With $\mathcal {C}^{\mathrm {E}}_{\mathrm {e}}$, particularly edges connecting to vertex 25 (Cologne) are highlighted. This is to be expected, as Cologne—besides being the most densely populated city in NRW—also records the highest commuter volume in NRW.

With $\mathcal {C}^{\mathrm {N}}_{\mathrm {e}}$, we observe edges to be important that are far off the expected commuter centers of NRW, namely the population-dense districts like vertex 25 (Cologne) as well as vertices in the Ruhr area. Certain peripheral edges (near the borders of NRW) are identified as important: for example, the edge connecting vertices 47 (Steinfurt) and 4 (Borken). These two urban districts do neither have a large population density nor a high commuter volume. The commuter traffic between them, however, is comparably large so that these two districts could be interpreted as one large district. Similar observations can me made for edges that connect vertices 50 (Viersen), 31 (Moenchengladbach) and 24 (Krefeld), vertices 40 (Rhein-Erft Kreis), 41 (Rhein-Kreis Neuss) and 42 (Rhein-Sieg Kreis), as well as for the edge that connects vertices 8 (Duisburg) and 12 (Essen) and the edge that connects vertices 19 (Herford) and 27 (Olpe).

### Evolving functional brain networks during an epileptic seizure

**Figure 5:**
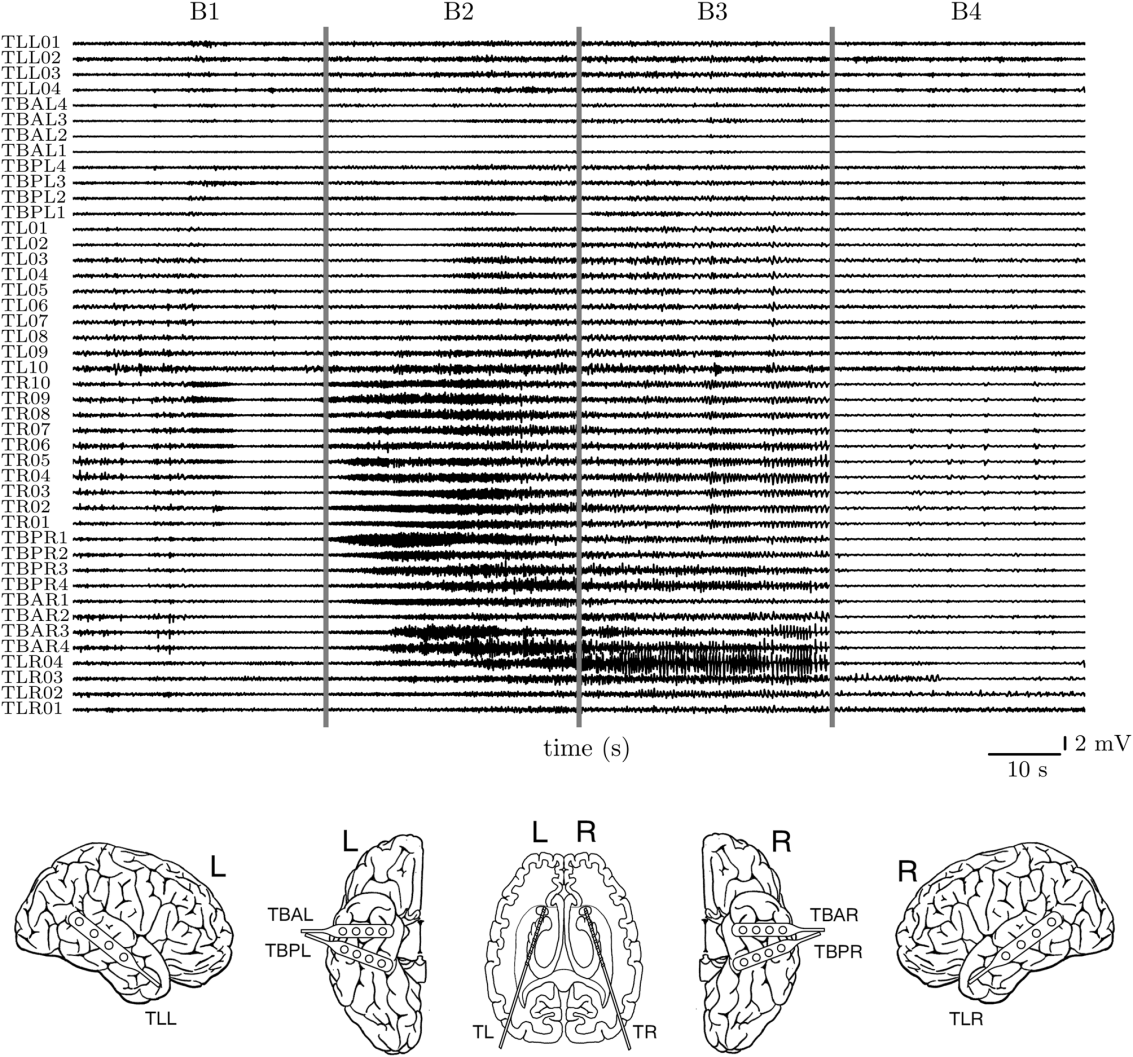
Brain dynamics during an epileptic seizure Top: invasive electroencephalographic recording of a seizure from the left (upper half) and right brain hemisphere (lower half). Block B1 indicates the pre-seizure phase; blocks B2 and B3 indicate the first and second half of the seizure (electroencephalographic seizure onset and ending determined automatically^70^); block B4 indicates the post-seizure phase. Bottom: schematics of sensors placed over the left and right temporal-lateral and temporal-basal neocortex and of bilateral intrahippocampal sensors.

Epilepsy is widely accepted as a large-scale network disease of the human brain^8,71^, and it is of utmost importance to not only identify central network vertices and characterize their dynamics but also to identify and characterize central edges in evolving functional brain networks. An improved characterization of time-dependent changes of centrality of network constituents could advance understanding of seizure generation, spread, and termination as well as could aid in the development of novel treatment options.

Here, we re-analyze evolving functional brain networks^42^ that were derived from multichannel electroencephalographic (EEG) data recorded from a subject with epilepsy prior to, during, and after a focal-onset seizure (see Fig. 5). The subject had signed informed consent that the clinical data might be used and published for research purposes. The study protocol had been approved by the ethics committee of the University of Bonn and is accordance with the tenets of the Declaration of Helsinki.

Briefly, the EEG data were recorded from sensors placed on the cortex and within relevant brain structures during the presurgical evaluation of the subject’s medically uncontrollable epilepsy. Evolving weighted functional brain networks were derived by associating vertices with the sampled brain regions (sensors) and edges represent the time-varying strength of interactions between pairs of brain regions. For the latter, a sliding-window approach was pursued (consecutive non-overlapping windows of 2.5 s duration each; corresponding to 500 data points) to calculate—in a time-resolved manner—the mean phase coherence *R*^72^, which is an established data-driven method for studying time-variant changes in phase synchronization in EEG time series. *R* is confined to the unit interval ($R=1$ indicates fully synchronized systems) and is taken as an estimate for the strength of interaction between a pair of brain regions. We refer the reader to Ref.^42^ for further details.

**Figure 6:**
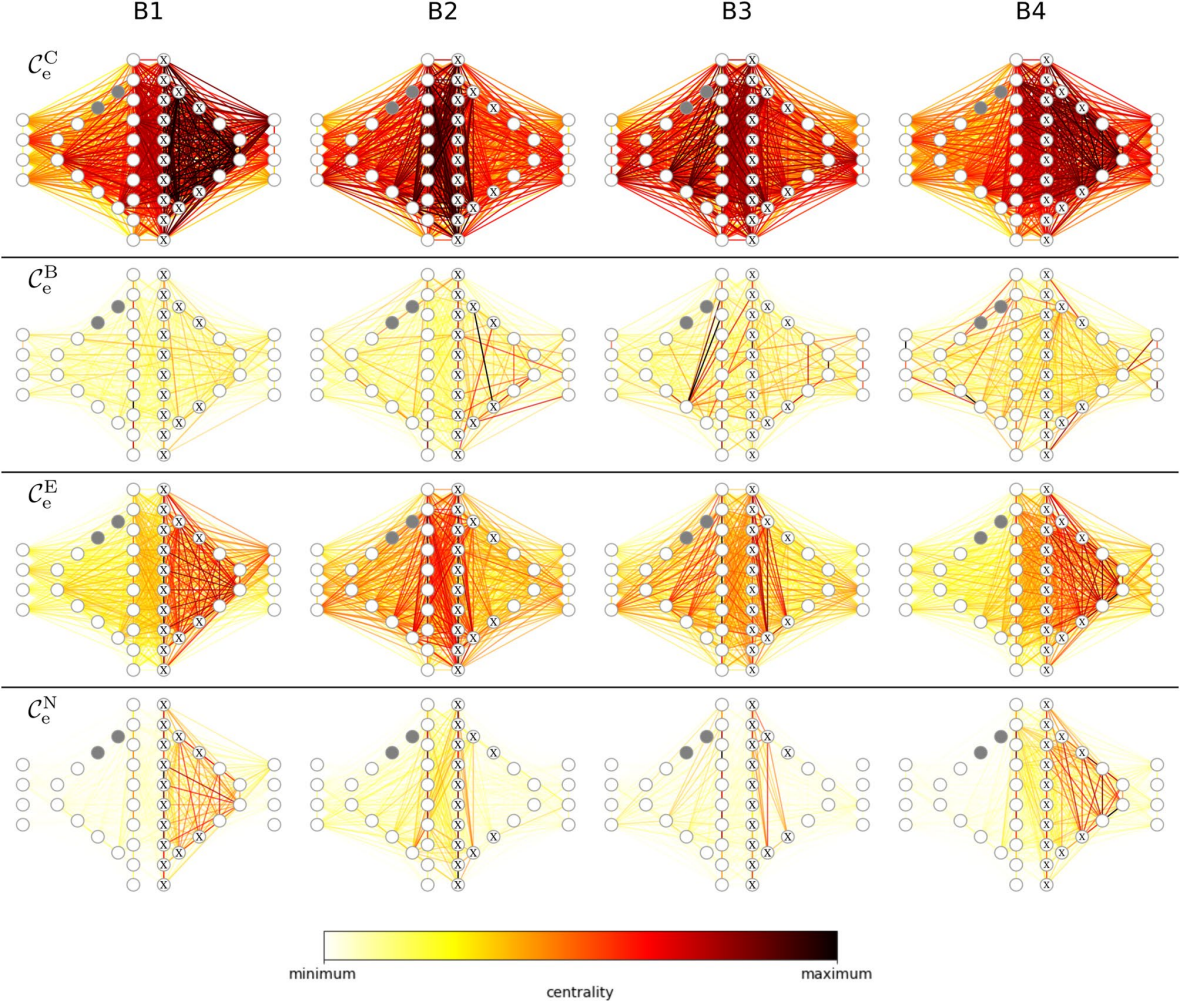
Key interactions in evolving functional brain networks during an epileptic seizure. Interactions identified with different edge centrality concepts (edge closeness centrality $\mathcal {C}^{\mathrm {C}}_{\mathrm {e}}$; edge eigenvector centrality $\mathcal {C}^{\mathrm {E}}_{\mathrm {e}}$; edge betweenness centrality $\mathcal {C}^{\mathrm {B}}_{\mathrm {e}}$; nearest-neighbor edge centrality $\mathcal {C}^{\mathrm {N}}_{\mathrm {e}}$). Vertices and edges projected onto a schematics of implanted sensors shown in Fig. 5. Edge color encodes normalized mean centrality values for each block (B1, $\dots$, B4). Vertices marked with ‘x’ record from the clinically defined seizure onset zone, and grey-colored vertices indicate EEG reference sensors (not included in analyses).

In Fig. 6, we present our findings that we obtained from investigating edge centrality in the temporal sequence of the weighted snapshot networks. Since centrality in such networks can vary strongly over time^60,73,74^, we partition the recording into four blocks (B1, $\dots$, B4) of equal duration. Each block contains the data from 13 consecutive snapshot networks and in the following, we report aggregated centrality values for each block.

For the evolving functional brain networks prior to and after the seizure (blocks B1 and B4), three of the four employed edge centrality concepts—namely $\mathcal {C}^{\mathrm {C}}_{\mathrm {e}}$, $\mathcal {C}^{\mathrm {E}}_{\mathrm {e}}$, and $\mathcal {C}^{\mathrm {N}}_{\mathrm {e}}$—yield qualitative similar findings. Edges connecting vertices related to the right brain hemisphere and particularly those that connect to vertices associated with the clinically defined seizure onset zone (located in the right brain hemisphere) excel with noticeably larger centrality values than edges connecting vertices related to the left hemisphere as well as than edges connecting vertices in both hemispheres. As with the other investigated real-world networks, we observe a rather peaked distribution of $\mathcal {C}^{\mathrm {C}}_{\mathrm {e}}$ values. Hence the differentiation between hemispheres is not as distinct as with $\mathcal {C}^{\mathrm {E}}_{\mathrm {e}}$ or $\mathcal {C}^{\mathrm {N}}_{\mathrm {e}}$. With $\mathcal {C}^{\mathrm {B}}_{\mathrm {e}}$, we observe only few edges with large centrality values. Prior to the seizure (block B1), these edges are confined to the left brain hemisphere and to a large extent connect vertices that face the seizure onset zone. After the seizure (block B4), $\mathcal {C}^{\mathrm {B}}_{\mathrm {e}}$ highlights some edges in the left and right brain hemisphere.

During the first half of the seizure (block B2), all concepts indicate high-centrality edges to connect vertices in the left and in the right brain hemisphere and particularly vertices associated with the seizure onset zone as well as its homologous regions in the opposite brain hemisphere. $\mathcal {C}^{\mathrm {C}}_{\mathrm {e}}$ (and to a lesser extent also $\mathcal {C}^{\mathrm {E}}_{\mathrm {e}}$) additionally highlights a larger number of high-centrality interhemispheric edges that dilutes during the second half of the seizure (block B3). In contrast, with $\mathcal {C}^{\mathrm {B}}_{\mathrm {e}}$ the amount of high-centrality interhemispheric edges even slightly increases during B3. $\mathcal {C}^{\mathrm {N}}_{\mathrm {e}}$ indicates high-centrality edges to connect nearby vertices in homologous regions in the opposite brain hemisphere during B3.

Although the employed centrality concepts mostly indicate different edges as important (as expected), our findings point to widespread, even interhemispheric interactions as highly relevant for seizure dynamics. Although these findings need to be validated on a larger database, they indicate that characterizing important edges in evolving functional brain networks can help to improve understanding of the complicated spatial-temporal dynamics of epileptic seizures.

## Conclusion

We introduced a novel edge centrality concept—nearest-neighbor edge centrality—that is defined in an analogous manner as the well-known and widely used vertex degree/strength centrality. By investigating possible relationships to other edge centralities (such as edge betweenness, edge closeness, and edge eigenvector centrality^30,42,46^) we could demonstrate the suitability of nearest-neighbor edge centrality for an identification of central edges in paradigmatic network models as well as in real-world networks from various scientific domains. Despite the expected conceptual similarities to either of the compared edge centralities, nearest-neighbor edge centrality provides additional and non-redundant information about the role edges play in a network. Moreover, nearest neighbor edge centrality can be computed much faster (up to a factor of 10) than path-based or edge-adjacency-matrix-based edge centralities, since—by definition—it depends solely on the distribution of vertex strengths.

Generally, we consider our nearest-neighbor edge centrality concept to be advantageous particularly in those situations were path-based or more global centrality concepts may have limited significance, e.g., for investigations of local spreading phenomena. The joint use of vertex degree/strength centrality and nearest-neighbor edge centrality could help to improve understanding the role vertices and edges play in the larger networks and thus to gain deeper insights into central but local network phenomena.

The definition of the nearest-neighbor edge centrality as proposed here is based on vertex strength and it thus allows investigations of undirected and weighted networks. Nevertheless, extensions to directed as well as to binary networks, to networks of networks^75^, multigraphs^76^, or hypergraphs^77^ can be achieved taking into account the total or in- and out-degree/strength of vertices. Such extensions might even lead to a modification of existing or formulation of novel concepts and measures that—in addition to degree-/strength distribution—also include the distribution of nearest-neighbor edge centrality values, to achieve a more complete characterization of a network.

Eventually, and with an eye on the analysis of real-world data, we expect new insights, by revisiting, extending and modifying network-based time-series analysis techniques such as visibility graphs^78^. We are confident that the nearest-neighbor edge centrality concept will help to improve characterization of networks through a data-driven identification of important edges.

## Data Availability

The data for this work was taken from the following sources: The Zachary’s karate club network data was taken from The KONECT Project (http://konect.cc/). The commuter traffic data was taken from data collected by the Statistisches Landesamt NRW (https://www.landesdatenbank.nrw.de/link/statistikTabellen/19321—Statistik: 19321). The rest of the data may be made available, upon request to the authors.

## Code availability

The code for the analysis was programmed using standard libraries in Python. All the calculations can be reproduced with the equations provided in the main text. Even so, the code used here is available upon request to the authors.
